# Acalculous Acute Cholecystitis in Previously Healthy Children: General Overview and Analysis of Pediatric Infectious Cases

**DOI:** 10.1155/2015/459608

**Published:** 2015-11-11

**Authors:** Dimitri Poddighe, Matteo Tresoldi, Amelia Licari, Gian Luigi Marseglia

**Affiliations:** ^1^Dipartimento di Pediatria, Azienda Ospedaliera di Melegnano, Via Pandina 1, Vizzolo Predabissi, 20070 Milano, Italy; ^2^Dipartimento di Chirurgia Generale, Azienda Ospedaliera di Melegnano, Via Pandina 1, Vizzolo Predabissi, 20070 Milano, Italy; ^3^Dipartimento di Pediatria, Fondazione IRCCS Policlinico San Matteo, Universita degli Studi di Pavia, Piazzale Golgi 19, 27100 Pavia, Italy

## Abstract

Acute acalculous cholecystitis (AAC) is an inflammation of the gallbladder, which does not appear to be associated with the presence of gallstones. AAC is estimated to represent more than 50% of cases of acute cholecystitis in the pediatric population. Although this pathology was initially described in critically ill patients, actually most pediatric cases have been observed during several infectious diseases. Particularly, here we reviewed pediatric infectious acute acalculous cholecystitis and analyzed the pathophysiological and clinical aspects of bacterial and viral forms.

## 1. Overview on Pediatric Acalculous Cholecystitis

Acute acalculous cholecystitis (AAC) is an inflammation of the gallbladder, which does not appear to be associated with the presence of gallstones. In childhood, AAC is the most frequent form of acute cholecystitis, differently from adult population, where the production of gallstones represents the main pathological mechanism inducing the disease. In adults, AAC represents only 5–10% of all cases of cholecystitis [1].

The existence of AAC has been recognized in the pediatric population for long time. In 1968, Marks et al. described two children affected with AAC and reported its prevalence during childhood as being around 30% cases of cholecystitis, in the medical literature of that time [2]. Actually, nowadays AAC is estimated to represent 50% to 70% of all cases of acute cholecystitis in the pediatric population and most cases were observed during infectious illnesses [3].

However, such disease was initially recognized in the clinical setting of the critically ill patient, after major surgery (especially cardiovascular interventions) or because of multiple trauma or extended burns. In these medical conditions, the prognosis of AAC is often concerning, as the overall mortality rate is estimated to be greater than 30% of cases. Here, AAC is often suspected because of the observation of plasma biochemical alterations suggesting cholestasis; then, the diagnosis is usually achieved by performing an abdominal ultrasonography and can be completed by the computerized tomography, as the therapy is usually represented by surgery, namely, cholecystostomy and/or cholecystectomy [1, 4, 5]. Indeed, in critically ill adult or pediatric patients, AAC often recognizes the gallbladder ischemia as a fundamental pathological aspect, which can be the consequence of several pathogenetic mechanisms. With regard to the pediatric age, Tsakayannis et al. described a cohort of 25 patients (observed between 1970 and 1994): most children developing AAC presented an underlying clinical condition, such as cardiac surgery or a systemic medical illness (e.g., leukemia, end-stage liver disease, leukemia, hemolytic uremic syndrome, and cystic fibrosis). Thus, the main risk factors for AAC in these children were prolonged fasting, total parenteral nutrition, intravenous opiate narcotics, volume depletion (shock), multiple transfusion, and sepsis [3]. The first three factors affect gallbladder depletion and, thus, lead to bile stasis; the other three aspects support the role of local hypoperfusion in the setting of systemic hypotension and/or volume depletion. Moreover, sepsis could promote gallbladder inflammation also through the production of proinflammatory and vasoactive mediators. Indeed, infectious AAC can arise in the context of a severe and systemic infection, leading to cardiovascular instability and, thus, hypoperfusion to several organs (including gallbladder) [4, 6].

Although AAC can occur in patients affected with AIDS or treated with immune-suppressive drugs after organ transplantation, actually most cases of pediatric AAC have been described in association with not acutely life-threatening infections in otherwise healthy patients, namely, without underlying diseases leading to the impairment of immune system or promoting the onset of acute complications. Many bacterial infections (such as leptospirosis, tuberculosis, bacterial enteritis, typhoidal or nontyphoidal salmonellosis, and brucellosis) have been reported as being causative of AAC, but a lot of pediatric cases have been described during viral illnesses too [4, 7, 8].

Whereas in acutely ill children the diagnostic suspicion of AAC often derives from the findings of biochemical abnormalities suggesting cholestasis and liver dysfunction, the main clinical manifestations of AAC in children being able to communicate their symptoms are the abdominal pain (mild to severe), being often more pronounced at the right upper quadrant (RUQ), and fever. These symptoms can be part of a variable clinical picture, including vomiting, diarrhoea, jaundice, and hepatosplenomegaly. Vomiting and jaundice are not always observed. Thus, the clinical presentation is quite unspecific and the diagnosis can be challenging, especially whenever AAC is superimposed to an acute hepatitis, whose clinical findings can be similar because of the frequent occurrence of concomitant intrahepatic cholestasis [9]. The diagnosis of AAC is usually obtained through abdominal ultrasonography (US), which can reveal the following findings: increased gallbladder wall thickness (>3.5–4 mm), pericholecystic fluid, and presence of mucosal membrane sludge. The presence of at least two of these aforementioned US criteria, in addition to the absence of gallstones, defines the diagnosis of AAC in the pediatric age [10].

In such clinical setting, namely, AAC arising in previously healthy children, where the diagnosis can be earlier than in acutely ill and noncommunicating patients and the etiology is primarily infectious, the clinical management is usually conservative. Here, identifying the infectious cause of AAC contributes to amelioration of the prognosis through an appropriate antibiotic treatment, especially if the etiology of the disease is bacterial [11].

Viral AAC have a better prognosis than bacterial forms and are usually managed by a conservative therapy only, based on the supportive treatment with intravenous fluid replacement, analgesics, and also antibiotics, until the viral etiology is confirmed [12]. Indeed, the causative infectious agent is not evident immediately and microbiological investigations need some days to be completed. Laboratory parameters and ultrasound findings do not allow distinguishing safely between bacterial and viral AAC. Bacterial AAC usually show leukocytosis and increased inflammatory indexes; however, these laboratory findings can be variably seen also in viral ACC and, therefore, blood cell count, plasma C-reactive protein, and other biochemical markers are not conclusive with regard to the etiology. Moreover, some alterations of liver enzymes, *γ*GT and ALP, are constantly described in all cases of AAC [6, 13].

## 2. Infectious Acute Acalculous Cholecystitis in Children

Through a revision of the medical literature regarding both adults and children, many infectious pathogens have been described in association with AAC, including bacteria (*Brucella* spp.,* C. jejuni*,* C. burnetii*,* Leptospira* spp.,* Mycobacterium* spp.,* Salmonella* spp., and* V. cholerae*), yeasts (*Candida* spp.), viruses (HAV, HBV, EBV, CMV, and* Flavivirus*), and parasites (*Plasmodium* spp.,* A. lumbricoides*, and* Echinococcus* spp.) [4].

According to the previous section, the common pathogenetic aspect of AAC appears to be a gallbladder injury, which can derive from (1) a direct trauma to the gallbladder, deriving from blunt abdominal trauma or from a surgical procedure (e.g., appendicectomy); (2) gallbladder ischemia, because of cardiovascular instability (e.g., cardiovascular surgery, congestive heart failure, and shock) or artery occlusion (e.g., vasculitis, diabetes mellitus); and (3) chemical injury from bile stasis, because of impaired emptying of gallbladder (e.g., absence of oral feeding, use of opiates, and cystic duct obstruction) [6].

Concerning the pathogenesis of infectious AAC, the aforementioned pathophysiological aspects involved in acutely ill patients, actually, are less evident or even absent in otherwise healthy children developing such a gallbladder disease. In these patients lacking predisposing factors, such as major surgery, multiple trauma and burn injury, primary infectious/inflammatory processes, and/or dehydration, are supposed to be variably involved in the pathogenesis of AAC [14, 15]. Severe volume depletion can lead to the concentration of bile, whose stasis can promote gallbladder mucosal injury, because of the content of some chemical compounds (e.g., lysophosphatidylcholine, *β*-glucuronidase) inside the dense and stagnant bile [4, 16, 17]. Infectious diseases involving the gastrointestinal system are often associated with dehydration, because of the vomiting and/or diarrhoea; moreover, sick children usually manifest a loss of appetite and the prolonged absence of eating impairs the emptying of the gallbladder, which contributes to the occurrence of cholestasis [6]. Hypoperfusion-related gallbladder ischemia is considered to be an important factor in the pathogenesis of ACC in critically ill and/or shocked patients, but that does not happen in healthy children [10]. Actually, some vasoactive mediators, produced during systemic or local infections, have been hypothesized to be involved in the pathogenesis of AAC [4, 18].

In 2002, Imamoglu et al. reported a personal AAC case series of 12 children, collected between 1990 and 2000. This is the first study where most children affected with AAC were not critically ill and, in fact, 75% patients were managed as outpatients requiring nonoperative treatment only. Interestingly, most patients of this cohort had a personal history of blunt abdominal trauma or surgery, but there were three children without any previous clinical issue, developing AAC in the setting of a common, but different, infectious illnesses, respectively pneumonia, acute gastroenteritis, and otitis media. This observation seems to account for heterogeneous (and largely unclear) mechanisms of disease, as discussed below [19].

Although an ischemic injury could be present in malaria-related AAC and some degree of hypoperfusion might occur during infectious diseases dominated by persistent emesis and/or volume depletion (e.g., cholera, enteric salmonellosis), actually bile stasis seems to be the main trigger factor of most infectious AAC in children [4, 15]. Extrinsic biliary obstruction was described in ascariasis and echinococcosis, but the pathogenetic mechanisms of AAC, arising during viral illnesses or nonseptic bacterial infections, remain still elusive, although portal lymphadenitis with extrinsic cystic duct obstruction has been hypothesized [4, 10]. Chirdan et al. described 16 previously healthy children (8–18 years) developing AAC. An infectious cause was detected in eight of them: 2 children were diagnosed as having malaria and* Salmonella typhi* infection was detected in 6 patients, through direct (2 patients had positive cultures from bile and blood) or indirect (4 children had a significant elevation of Widal titer) microbiological methods. Actually, authors reported the blockage of the cystic duct by an enlarged lymph node in one patient only [20]. Similarly, in the study by Gnassingbé et al., including 6 children with gangrenous or perforated AAC, where the histological features of gallbladder were described, no distal extrinsic compression on the biliary duct was reported [21].

Recently, important insights contributed to the understanding of the pathogenesis of gallbladder diseases during typhoid fever. Although the infectious process of* S. typhi* passes through the infection of mesenteric nodes before getting into the blood stream, actually the bacteria showed a tropism for the epithelium of the gallbladder: in animal models, the bacterial appearance in the gallbladder has been shown to anticipate the detection in the intestine, suggesting that the gallbladder colonization by* S. typhi* is not an ascending process from the gastrointestinal tract. Here, the bacterial colonization is able to trigger a strong inflammatory response with high local level of proinflammatory cytokines, which causes an important neutrophil infiltration leading to the damage of the vesicular wall [22]. Such local inflammatory injury might be involved also in AAC caused by other bacteria, especially in the setting of septicemia, where a bacterial direct localization in the gallbladder is more plausible; of course, most bacteria do not have the same gallbladder tropism as* S. typhi*, but a concomitant injury, by instance because of the occurrence of cholestasis and/or ischemia, could promote a bacterial superinfection [23].

Recently, Lee et al. analyzed retrospectively 67 children diagnosed as having AAC. Fewer than 40% of cases occurred in patients affected with underlying systemic diseases or specific diseases with liver involvement and 36% cases were caused by systemic bacterial infections (*S. typhi*,* S. pyogenes*,* A. baumannii*,* S. pneumoniae*,* S. epidermidis*,* B. cepacia*, and* S. maltophilia*), where a gallbladder localization from bloodstream could be supposed. Interestingly, 27% of AAC cases appeared in the clinical setting of acute hepatitis [12].

This finding is evident also in our analysis of AAC pediatric case reports, representing the main type of publication on AAC in children since 2000. In Table 1, we summarized all available English-written papers describing cases of infectious ACC in previously healthy children [11, 24–47]. These reports do not consider children with underlying autoimmune diseases (e.g., systemic lupus erythematous, Kawasaki disease), chronic diseases (e.g., cystic fibrosis), and primary or secondary immunodeficiency syndromes (e.g., AIDS, chronic granulomatous diseases), which were excluded. Thus, 27 pediatric ACC case reports have been found. Interestingly, most dealt with viral illnesses, particularly HAV and EBV infections, which are well-known causes of acute hepatitis. Those accounted for 14 cases, representing around the half of the totality. Thus, viral hepatitis seems to represent the main setting where pediatric AAC develops. Excluding two cases of suspected, but unknown, infectious etiology, the others were due to* Plasmodium* spp. (5),* Salmonella* spp. (2),* S. aureus* (1),* V. cholerae* (1),* C. burnetii* (1), and* B. melitensis* (1). Actually, Q-fever associated AAC was supposed to be mediated by the presence of anti-phospholipid antibodies, which are commonly observed in adult Q-fever and could be responsible for some atypical manifestations [43]. On the contrary, brucellosis is a zoonotic infection characterized by bacterial replication in the lymph nodes near to the site of penetration, followed by bacteremia. AAC is a very rare complication of brucellosis and the analysis of adult cases showed the isolation of bacteria in the gallbladder with signs of both chronic and acute inflammation [48, 49].

In the absence of sufficient pathologic data, because of the good prognosis of viral AAC (which very often allows a conservative clinical management), the pathogenesis of these forms has been poorly understood. However, it is known that viral hepatitis can show cholestatic aspects, which means that, in addition to the presence of more pronounced jaundice and elevation of ALP and *γ*GT, the bile production and composition can be altered at the hepatocyte level. Bile secretion is warranted by a set of hepatocanalicular proteins transporting biliary lipids into the bile canaliculi [50]. Genetic polymorphisms of these transporters of phosphatidylcholine, bile salts, and sterols, and the acute viral infection of hepatocytes might interplay, leading to intrahepatic cholestasis [51].

Interestingly, more than 30% of cases of infectious AAC (recorded in Table 1) appeared to be associated with EBV. Most part of viral AAC has been described in last few years, which could be due to an increase of the diagnostic sensitivity, as maybe ultrasonography has been used more often during acute liver diseases. Indeed, as well as in HAV infection, cholestasis is a common clinical finding in EBV hepatitis too: this could be related to the local and systemic production of inflammatory cytokines, which can alter the functioning of canalicular transporting systems [52]. Inflammation-induced cholestasis, caused by intrahepatic and extrahepatic infections as well as hepatotoxic drugs, has been suggested to derive from the impairment of several membrane transporters regulating bile salt and acids, which can lead to several abnormalities in bile composition and concentration and, finally, could damage the gallbladder wall. Thus, viral cholestatic hepatitis, both in HAV and EBV infection, could induce gallbladder inflammation, namely, AAC, through these mechanisms, although further studies are needed to elucidate the exact pathogenesis [53, 54]. Interestingly, two-thirds of pediatric patients reported as being affected by viral AAC, in our case reports review, were female: sex-related differences in the activity of enzymatic systems involved in bile synthesis and transport might predispose to intrahepatic cholestasis during viral hepatitis and, thus, might promote the onset of AAC with a greater frequency in females than in males.

## 3. Conclusion

In childhood, AAC is the most frequent form of acute cholecystitis. In otherwise healthy and not critically ill children, most cases of AAC are related to infections. Many infectious agents have been described as a cause of AAC, but viruses seem to be the most represented in the recent reports. Differently from AAC arising during some bacterial and/or septic infections and in critically ill or surgical patients, viral AAC have a good prognosis, as the clinical management is almost always conservative. In the pediatric age, viral AAC has been mainly associated with HAV and EBV hepatitis: here, the concomitant intrahepatic cholestasis could cause some alterations of the concentration and the composition of the bile, and the stasis of such an altered bile in the gallbladder might induce a mucosal injury of vesicular wall, leading to acute inflammation, namely, AAC.

## Figures and Tables

**Table 1:** Case reports of pediatric AAC in previously healthy children since 2000.

Authors (year)	Age	Sex	Etiology	Symptoms	Reference
Ashley et al. (2000)	4	M	*B. melitensis*	RUQ pain, fever, constipation, anorexia	[24]
Ciftci et al. (2001)	7	M	HAV	Abdominal pain, fever, jaundice	[25]
Croteau et al. (2001)	2	M	N.A.	Abdominal pain, fever, anorexia	[26]
Lo et al. (2002)	5	M	*Salmonella* spp. (group D)	Abdominal pain, fever, vomiting, diarrhoea	[27]
Batra et al. (2003)	12	M	*S. aureus*	RUQ pain, fever, jaundice, maculopapular rash	[28]
Garel et al. (2003)	4	M	N.A.	Abdominal pain, fever, vomiting, diarrhoea	[29]
Saha et al. (2005)	7	F	*P. falciparum*	RUQ pain, fever	[30]
Axelrod and Karakas (2007)	3	F	*S. typhi*	Abdominal pain, fever, vomiting	[31]
Kuttiat and Kohli (2007)	8 and 9	Both M	*P. falciparum* and *P. vivax*	RUQ pain, fever, vomiting (*P. vivax*)	[32]
Lagona et al. (2007)	4	F	EBV	RUQ pain, fever, jaundice, vomiting, anorexia	[33]
Anthoine-Milhomme et al. (2007)	7	F	*Plasmodium* spp.	Abdominal pain, fever, diarrhoea, jaundice	[34]
Prassouli et al. (2007)	13	F	EBV	Abdominal pain, fever, vomiting, jaundice	[35]
Gora-Gebka et al. (2008)	9 and 4	Both F	EBV + CMV and EBV	RUQ pain, fever, jaundice, enlargement of liver and spleen	[36]
Kumar et al. (2008)	3	F	*P. falciparum*	Abdominal pain, fever, vomiting	[37]
Attilakos et al. (2009)	5	M	EBV	Fever, jaundice, enlargement of liver and spleen	[38]
Suresh et al. (2009)	2	F	HAV	Abdominal pain, fever, vomiting	[39]
de Souza et al. (2009)	16	M	HAV	Abdominal pain, fever, vomiting	[40]
Arroud et al. (2009)	11	M	HAV	Abdominal pain, fever, vomiting, jaundice	[41]
Herek et al. (2011)	9	M	HAV	Abdominal pain, fever, vomiting, jaundice	[42]
Newcombe et al. (2013)	9	M	*C. burnetii*	N.A.	[43]
Poddighe et al. (2014)	7	F	EBV	RUQ, fever, vomiting, jaundice, liver enlargement	[11]
Kim et al. (2014)	10	F	EBV	RUQ pain, fever, cervical lymphadenopathy	[44]
Fretzayas et al. (2014)	11 and 12	Both F	EBV	Abdominal pain, fever, jaundice, hepatosplenomegaly	[45]
Alkoury et al. (2015)	15	F	EBV	Abdominal pain, fever, vomiting	[46]
Pawlowska-Kamieniak et al. (2015)	17	F	EBV	RUQ pain, fever, anorexia	[47]
